# Somatic Host Cell Alterations in HPV Carcinogenesis

**DOI:** 10.3390/v9080206

**Published:** 2017-08-03

**Authors:** Tamara R. Litwin, Megan A. Clarke, Michael Dean, Nicolas Wentzensen

**Affiliations:** 1 Cancer Prevention Fellowship Program, Division of Cancer Prevention, National Cancer Institute, Rockville, MD 20850, USA; tamara.litwin@nih.gov (T.R.L.); megan.clarke@nih.gov (M.A.C.); 2 Clinical Genetics Branch, Division of Cancer Epidemiology and Genetics, National Cancer Institute, Rockville, MD 20850, USA; 3 Laboratory of Translational Genomics, Division of Cancer Epidemiology and Genetics, National Cancer Institute, Gaithersburg, MD 20850, USA; deanm@mail.nih.gov

**Keywords:** HPV, somatic mutation, cervical cancer, APOBEC, significantly mutated gene, copy number variation, chromosomal instability, head and neck cancer, integration

## Abstract

High-risk human papilloma virus (HPV) infections cause cancers in different organ sites, most commonly cervical and head and neck cancers. While carcinogenesis is initiated by two viral oncoproteins, E6 and E7, increasing evidence shows the importance of specific somatic events in host cells for malignant transformation. HPV-driven cancers share characteristic somatic changes, including apolipoprotein B mRNA editing catalytic polypeptide-like (APOBEC)-driven mutations and genomic instability leading to copy number variations and large chromosomal rearrangements. HPV-associated cancers have recurrent somatic mutations in phosphatidylinositol-4,5-bisphosphate 3-kinase catalytic subunit alpha (*PIK3CA*) and phosphatase and tensin homolog (*PTEN*), human leukocyte antigen A and B (*HLA-A* and *HLA-B*)*-A/B*, and the transforming growth factor beta (TGFβ) pathway, and rarely have mutations in the tumor protein p53 (*TP53*) and RB transcriptional corepressor 1 (*RB1*) tumor suppressor genes. There are some variations by tumor site, such as *NOTCH1* mutations which are primarily found in head and neck cancers. Understanding the somatic events following HPV infection and persistence can aid the development of early detection biomarkers, particularly when mutations in precancers are characterized. Somatic mutations may also influence prognosis and treatment decisions.

## 1. Introduction

High-risk human papilloma virus (HPV) infections cause cancers at many sites. It is estimated that almost all cervical cancers [1], 20–70% of oropharyngeal cancers and 5–30% of other head and neck cancers [2,3,4], 88% of anal cancers [5], 48% of penile cancers [6,7], 19% of vulvar cancers [5], and 71% of vaginal cancers [5] are caused by HPV, with some geographic variation observed for the non-cervical cancers. Together, these cancers resulted in approximately 610,000, or 5%, of all cancer diagnoses worldwide in 2008 [8,9]. 

HPV infection alone is an insufficient cause of carcinogenesis. Most HPV infections become undetectable after a few months and never result in malignancies, with 91% becoming undetectable after two years, although it has been proposed that there may be some level of persistent latent infection that is undetectable by PCR [10,11]. High-risk HPV types persist longer on average than low-risk types [12]. A failure to clear the virus results in viral persistence, but many persistent infections never develop into precancerous lesions [13]. Finally, even advanced precancerous cervical intraepithelial neoplasias grade 3 (CIN3) only progress to invasive cancer in 30% of cases over 30 years [14]. When infections persist over time, somatic mutations may accumulate and contribute to the development of precancerous lesions, and then finally to malignant cancers. Understanding the complete carcinogenic pathways is important for developing new strategies to prevent HPV-associated cancer mortality, both through early detection and through targeted therapies [15,16].

HPV-derived cancers share many carcinogenic features across cancer sites, suggesting that the viral oncoproteins E6 and E7 work similarly at different sites. A previous review on this topic [17] predates recent publications of large genomic data from HPV-driven cervical and head and neck cancers in The Cancer Genome Atlas (TCGA) [2,18]. Here, we review common somatic mutations, copy number alterations, and related pathways identified by TCGA and other recent efforts. While the focus of this review is on somatic changes, genome-wide association (GWAS) studies of cervical and HPV-related head and neck cancers have shown that there is also a heritable component. At both cancer sites, human leukocyte antigen (HLA) variants are among the few consistent, independently replicated findings from GWAS studies [19,20,21].

## 2. Mechanisms of HPV-Mediated Mutagenesis

**Figure 1:**
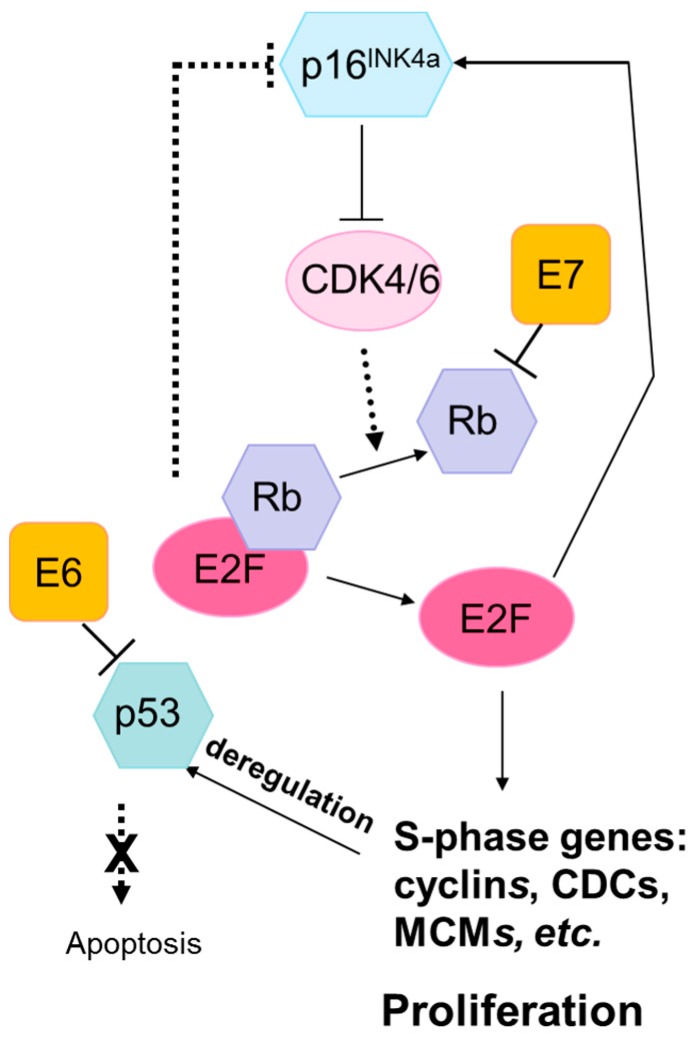
The Rb and p53 pathways are disrupted by the human papilloma virus (HPV) oncoproteins E7 and E6, respectively. The HPV E7 protein binds to Rb with high affinity, disrupting its interaction with the transcription factor E2F. This results in the release and activation of E2F, driving expression of S-phase genes and cell cycle progression. P16^INK4A^ is a cyclin-dependent kinase inhibitor that regulates the cell cycle by inactivating cyclin-dependent kinases involved in Rb phosphorylation. Upregulation of p16^INK4A^ is induced by HPV-mediated disruption of E7, leading to the accumulation of p16^INK4A^ in HPV-transformed cells. The HPV E6 protein inhibits apoptosis by targeting the tumor suppressor protein, p53, for degradation. HPV E6 inhibition of p53 promotes cell proliferation and can lead to genomic instability and the accumulation of somatic mutations. Abbreviations: Rb, retinoblastoma protein; p16^INK4A^, cyclin-dependent kinase inhibitor 2A; CDK, cyclin-dependent kinases; E2F, E2F transcription factor; CDC, cell-division-cycle genes; MCM, minichromosome maintenance family.

There is a great diversity of HPV genotypes, but only a small subset is carcinogenic; among these, HPV16 alone accounts for 50–90% of HPV-driven cancers depending on the site, with some regional variations [22,23]. Most cancers evaluated in studies included in this review are caused by HPV16, and there may be variations in somatic mutation load and type by HPV genotype that are currently not adequately captured. Two of the eight proteins encoded by the HPV genome, E6 and E7, account for most carcinogenic effects of high-risk HPV types [15]. They promote carcinogenesis in several ways, including creating genomic instability and inhibiting tumor suppressor genes. E6 and E7 directly promote genomic instability, which can result in large chromosomal rearrangements and copy number variations, by interfering with centromere duplication during mitosis [24,25]. Both oncoproteins interfere with important cellular tumor suppressor pathways: E6 inhibits the p53 tumor suppressor by promoting its proteasomal degradation [26,27], while E7 disrupts the retinoblastoma (Rb) pathway resulting in uncontrolled activation of the cell cycle and induction of p16^INK4A^, a cyclin-dependent kinase inhibitor, through a disrupted feedback loop (Figure 1) [28,29,30]. Theoretically, since HPV oncoproteins are important carcinogenic drivers interfering with several cellular pathways, it could be expected that fewer somatic alterations are required for malignant transformation in HPV-associated compared to non-HPV associated cancers. There is some evidence of lower mutation load in HPV-positive compared to HPV-negative penile cancers [31]. However, the evidence is inconclusive for head and neck cancers, with one study showing evidence of a reduced somatic mutation load in HPV-positive compared to HPV-negative cancers [3] while the TCGA head and neck study did not find evidence of a difference [2].

In addition to direct viral effects, specific mutation signatures may be overrepresented in HPV-positive cancers due to host–viral interactions. The apolipoprotein B mRNA editing catalytic polypeptide-like (APOBEC) mutation signature in particular is very common in HPV-positive cancers, likely triggered by the host response to HPV infection [32]. 

### 2.1. Genomic Instability

**Figure 2:**
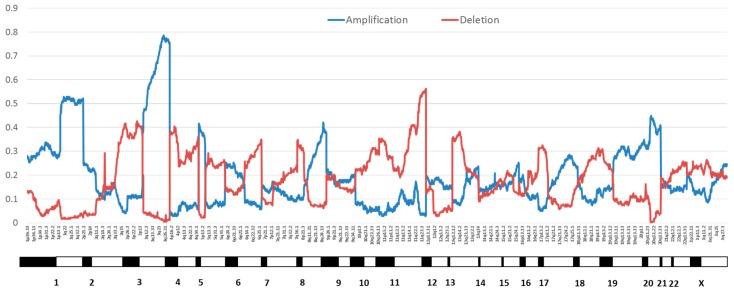
Proportion of cervical cancers with copy number variation by chromosome position from The Cancer Genome Atlas (TCGA) cervical cancer data [18]. Amplifications are in blue and deletions are in red.

Rates of copy number alterations vary across cancer sites. Cervical cancers average 88 copy number alterations in the TCGA dataset, including 26 amplifications and 37 losses [18]. Focal amplifications of loci containing genes discussed elsewhere in this review in order of frequency include 3q28 (tumor protein p63 (*TP63*), altered in 77% of samples), 3q24.1 (transforming growth factor beta receptor 2 (*TGFBR2*), 36%), 10q23.31 (phosphatase and tensin homolog (*PTEN*), 31%), 18q21.2 (SMAD family member 4 (*SMAD4*), 28%), and 7p11.2 (epidermal growth factor receptor (*EGFR*), 17%) [18]. Greater numbers of copy number variations were reported in cervical squamous cell carcinomas than in cervical adenocarcinomas [18]. A review of cervical squamous cell carcinomas from other datasets as well as limited information on HPV-positive vulvar squamous cell carcinomas also showed gains at 3q (55%), losses at 3p (36%), and losses at 11q (33%) [33]. A study of CIN3 lesions and invasive cancers reported an average of 36.3 copy number alterations in cancers, with the most frequent amplification at 3q (50% of cancers and 25% of CIN3) [34]. Notably, this region contains the phosphatidylinositol-4,5-bisphosphate 3-kinase catalytic subunit alpha (*PIK3CA*) gene, which is the most commonly mutated gene in HPV-driven cancers across sites (see below). Losses were most common in 3p (40% of cancers and 10% of CIN3) [34]. A summary of copy number alterations reported in HPV-driven cancers can be found in Table 1. Figure 2 shows the frequency of chromosomal amplifications and deletions across the whole genome in cervical cancers from TCGA [18].

In HPV-positive head and neck cancers, significant copy number losses have been reported in 22 genes and gains in 65 genes, including RB transcriptional corepressor 1 (*RB1*) and *PIK3CA* [37]. The 3q26-28 region is amplified in both HPV-positive and HPV-negative cancers, while 3p deletions are primarily found in HPV-negative head and neck cancers [37]. 

In penile cancers, greater copy number gains in 15 regions and losses in four regions are seen in HPV-positive compared to HPV-negative cancers [38]. Autosomal copy number variations are most frequently observed on chromosomes 3 and 8, including losses in 3p and gains in 3q, and are also associated with worse prognosis [38]. A small study of HPV-positive anal cancers reported recurrent gains in 17q, 3q, 19p, and 19q [39].

In HPV-driven cancers of the cervix and head and neck, copy number variations often co-localize with sites of viral integration [2,18], a phenomenon that occurs in many HPV-associated cancers, and has been shown to vary by HPV type [40,41,42]. Though the mechanisms by which HPV integrates into the host cell genome are not well understood, these events tend to occur at regions of genomic instability [34,42,43,44,45]. It has been proposed that copy number alterations commonly occur in regions of genomic instability, which in turn may promote viral integration in those locations, explaining why viral integration is more common at sites with copy number alterations than expected by chance [34]. Viral integration has also been observed in short regions of HPV and host genome sequence homology (i.e., “micro-homologies”), suggesting a potential role for DNA repair processes to integrate HPV and host cell genomes based on nucleotide sequence similarities [45,46].

Recurrent large chromosomal rearrangements have been reported in 23 locations in cervical cancers in TCGA [18]. One notable recurrent rearrangement is the 16p13 zinc finger CCCH-type containing 7Abreast cancer anti-estrogen resistance 4 (*ZC3H7A-BCAR4*) fusion, which together with copy number gain of the locus containing *BCAR4* (16p13.13, found in 20% of tumors) and duplication detected by whole genome sequencing suggest a potential role of this gene in cervical carcinogenesis [18].

HPV-driven cancers of the cervix, head and neck, and penis share copy number alteration sites, most notably copy number gains in 3q, which in addition to *PIK3CA* contains the telomerase RNA component (*TERC*), MDS1 and EVI1 complex locus (*MECOM*), SRY-box 2 (*SOX2*), and *TP63* genes [18,34,37,38]. It is worth noting that both HPV-positive and HPV-negative cancers display recurrent focal amplifications of this region [2]. Together with the extremely high somatic mutation rate of *PIK3CA* (see Section 3.2), this supports an important role for *PIK3CA* in HPV-mediated carcinogenesis.

### 2.2. Mutational Signatures

#### 2.2.1. APOBEC

The APOBEC family of cytosine deaminases causes cytosine to thymine or guanine mutations [47,48,49]. APOBEC3B, a subclass of these proteins, causes characteristic mutations that are enriched in many cervical and head and neck cancers [18,35,50,51,52]. During DNA repair, APOBEC-mediated cytosine deamination can result in characteristic mutational signatures that occur at motifs involving a thymine immediately 5′ to the target cytosine, collectively referred to as “TCW” mutations, where W corresponds to an A or T [52]. APOBEC-mediated mutagenesis is also enriched in HPV-positive subsets of many head and neck cancers [53] as well as in penile cancers [54] suggesting the activation of APOBEC enzymes in HPV-driven cancers across sites. 

**Figure 3:**
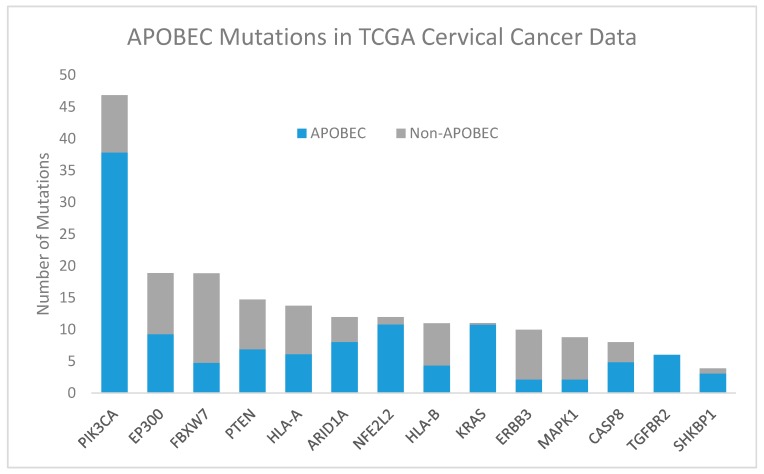
Apolipoprotein B mRNA editing catalytic polypeptide-like (APOBEC, blue) and non-APOBEC (gray) mutations in significantly mutated genes in TCGA cervical cancer data [18]. Abbreviations: PIK3CA, phosphatidylinositol-4,5-bisphosphate 3-kinase catalytic subunit alpha; EP300, E1A binding protein p300; FBXW7, F-box and WD repeat domain containing 7; PTEN, phosphatase and tensin homolog; HLA-A, human leukocyte antigen A; NFE2L2, nuclear factor, erythroid 2 like 2; ARID1A, AT-rich interaction domain 1A; HLA-B, human leukocyte antigen B; KRAS, KRAS proto-oncogene, GTPase; ERBB3, erb-b2 receptor tyrosine kinase 2; MAPK1, mitogen-activated protein kinase 1; CASP8, caspase 8; TGFBR2, transforming growth factor beta receptor 2; SHKBP1, SH3KBP1 binding protein 1.

APOBEC-associated mutations are responsible for many mutations of genes in the HPV-associated carcinogenesis pathways discussed below, including common *PIK3CA* point mutations [53]. APOBEC signature enrichment was reported in 150 of 192 exomes in TCGA cervical cancer data, with the fraction of ABOPEC signature mutations by gene reproduced in Figure 3 [18].

The APOBEC pathway drives mutations in many cancer sites including cervix, head and neck, bladder, lung, and breast [51,52]. However, APOBEC mutations are likely enriched in HPV-positive cancers due to its role in the host response to the viral infection. The APOBEC3A protein may inhibit HPV infectivity, so upregulation assists in viral clearance and reduces persistence [32], although it has also been suggested that APOBEC3B is likely to be the primary APOBEC involved HPV-related carcinogenesis because unlike APOBEC3A it is expressed in the nucleus [51]. The APOBEC mutagenesis pathway has also been reported to be upregulated by the HPV oncoprotein E6 [55]. Upregulation of APOBEC proteins in response to viral infection can cause “collateral damage” to the host DNA [56]. However, the exact mechanism of induction of the APOBEC pathway and its contribution to carcinogenesis once activated remain unclear, since it is also found in many cancer types not associated with infectious agents, including breast cancer and ovarian serous carcinoma [57,58,59]. Due to insufficient data from cancer precursors, it is currently not clear at what stage in the carcinogenic process APOBEC mutations start to accumulate and whether APOBEC mutations occur before non-APOBEC mutations.

#### 2.2.2. Other Mutational Signatures

Cervical cancer, which has an attributable risk for HPV of close to 100% [1], has two primary mutational signatures, classified as signature 1B and signature 2 by Alexandrov et al. [50]. Signature 2 is the above-discussed APOBEC signature. Signature 1B is a common pattern across many cancer sites that is characterized by cytosine to thymine mutations at methylated cytosine-guanine (CpG) sites along the DNA and is associated with age [50]. Other cancers associated with signature 1B include head and neck, the only other HPV-associated cancer characterized by this study, as well as ovarian and endometrial, the other major gynecological cancers [50].

## 3. Genes and Pathways

Many somatic mutations overlap across HPV-associated cancer sites. Frequently somatically mutated genes are summarized in Table 2. In the following sections, common mutations are discussed in the context of their respective pathways.

### 3.1. Lack of Mutations in TP53 and RB1

The HPV oncogenic proteins E6 and E7 target the tumor suppressor proteins p53 and pRB, respectively, for degradation [79]. They therefore obviate the need for somatic deactivation of the *TP53* and *RB1* genes during the carcinogenesis process, and mutations in these genes infrequently occur in HPV-positive cancers compared to corresponding HPV-negative cancers at the same sites (Figure 1). 

In cervical squamous cell carcinoma, *TP53* mutations have been reported with a frequency of 5% [35]. Although fewer than 1% of cervical squamous cell carcinomas are HPV-negative, one study reported a difference in *TP53* mutation status by classifying tumors in the TCGA-CESC data set as “HPV active” (expressing HPV transcripts; 4% TP53 mutation rate) versus “HPV inactive” (not expressing HPV transcripts; 47% *TP53* mutation rate and 8% of the total number of HPV-positive samples) [80]. This is consistent with the idea that TP53 inactivation is exceedingly common, and that the TP53 mutation rates are negatively correlated with HPV activity. Vulvar squamous cell carcinoma has an 8–16% TP53 mutation prevalence in HPV-positive tumors versus 30–76% prevalence in HPV-negative tumors, and vulvar intraepithelial neoplasia (VIN) precancerous lesions have a 3% *TP53* mutation prevalence in HPV-positive and 21% prevalence in HPV-negative lesions [73,74]. Likewise, TP53 mutations appear to be more prevalent in HPV-negative than in HPV-positive penile squamous cell carcinomas [31].

Numerous studies have reported significantly higher *TP53* mutation rates in HPV-negative (52–86%) compared to HPV-positive (0–25%) head and neck tumors [2,3,36,37,72]. A complete absence of *TP53* mutations in tumors with high-risk HPV types present has also been found in laryngeal [81] and esophageal [82] cancers. It has been suggested that *TP53* inactivation, either through HPV infection or somatic mutation, is nearly ubiquitous in head and neck squamous cell carcinomas, even those that are HPV-negative and therefore must achieve this inactivation via other pathways [3].

Head and neck cancers with wild-type *TP53* have a better prognosis than those with *TP53* mutations [2,83]. HPV positivity and p16 ^INK4A^ expression, which are both related to retention of wild type *TP53*, are also positively correlated with overall 3-year survival in anal cancers [84]. Evidence in penile cancers is mixed [85,86,87,88]. 

The Rb pathway controls the cell cycle and regulates growth and proliferation [89]. *RB1* mutations are very rare in cervical cancers because HPV E7 activity inactivates Rb tumor suppression activity by disrupting its interaction with the transcription factor E2F, making mutations in this gene unnecessary in HPV-positive cancers [90,91]. *RB1* is mutated in 6–24% of HPV-positive head and neck cancers, a similar fraction to HPV-negative head and neck cancers (4%) [2,36,37]. Cyclin dependent kinase inhibitor 2A (*CDKN2A*) encodes p16^INK4A^, an Rb pathway gene which as described above is nearly ubiquitously expressed in HPV-positive cancers due to activation of a negative feedback loop triggered by E2F release [92,93]. Overexpression of p16^INK4A^ is also common in HPV-related precancers, which has led to development of p16^INK4A^-based biomarkers for cervical cancer screening and triage [94,95]. *CDKN2A* is rarely altered in HPV-positive (0%) compared with HPV-negative head and neck cancers (25% mutation rate, frequent alterations in 9p21.3 chromosomal region containing the *CDKN2A* gene) [2]. An absence of *CDKN2A* alterations in HPV-positive penile squamous cell carcinomas has also been reported, compared with 16% mutation prevalence and 24% copy number reduction in HPV-negative tumors [31]. 

### 3.2. PI3K/AKT Pathway

**Figure 4:**
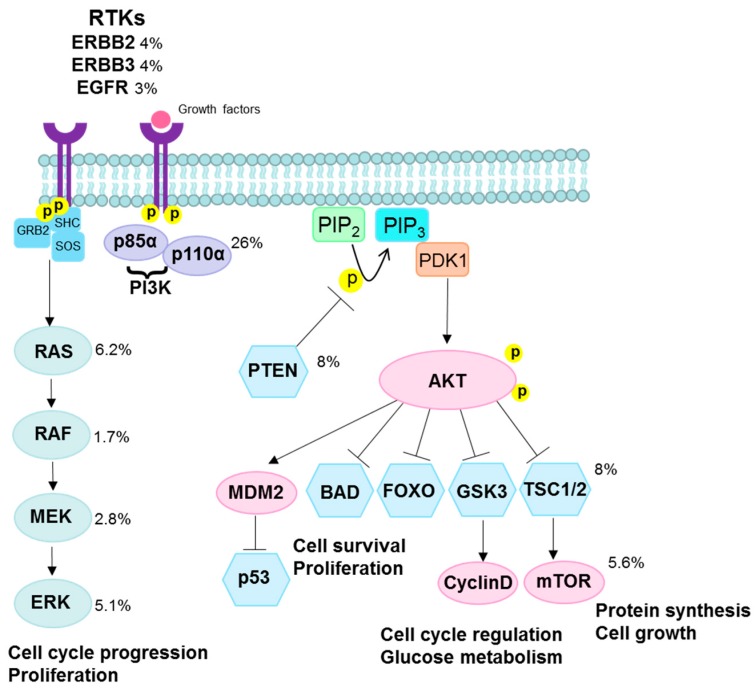
PI3K/AKT and RAS/EGFR/ERK pathways. Class IA PI3K are heterodimers consisting of a p85α regulatory subunit and a p110α catalytic subunit (encoded by PIK3CA). The p85α regulatory subunit normally stabilizes p110α and inhibits its catalytic activity. Activation of the PI3K pathway via ligand binding to transmembrane RTKs such as EGFR, ERBB2, and ERBB3, results in phosphorylation of p85α and activation of the p110α catalytic subunit. Once activated, PI3K phosphorylates PIP_2_ at the plasma membrane to produce the lipid second messenger, PIP_3_. This step is inhibited by PTEN, which dephosphorylates PIP_3_ to PIP_2_. PIP_3_ binds to PDK1 which phosphorylates and activates AKT. Activated AKT phosphorylates TSC1/2, leading to mTOR activation and increased protein synthesis and cell growth. AKT increases cell proliferation by phosphorylating GSK3 which normally regulates the degradation of cyclin D. In addition, activation of AKT promotes cell survival by inhibiting proapoptotic factors such as BAD and FOXO transcription factors, and by phosphorylating MDM2 which antagonizes p53-mediated apoptosis. Other PI3K activation pathways depend on adaptor proteins such as GRB2, which binds to and activates SOS, stimulating RAS and independent activation of p110α. A Ras-binding domain in p110α also mediates activation by RAS. RAS-mediated recruitment to the plasma membrane activates RAF, which in turn activates MEK and ERK, respectively. ERK phosphorylates several proteins that control cell proliferation and cell cycle progression. Somatic mutation frequencies in cervical squamous cell carcinomas are shown next to each gene [18]. Abbreviations: RTKs, receptor tyrosine kinases; ERBB2, erb-b2 receptor tyrosine kinase 2; ERBB3, erb-b2 receptor tyrosine kinase 3; EGFR, epidermal growth factor receptor; PI3K, phosphatidylinositol 3-kinases; AKT, protein kinase B; mTOR, mammalian target of rapamycin; PTEN, phosphatase and tensin homolog; PIK3CA, phosphatidylinositol 3-kinase catalytic subunit alpha; PDK1, phosphoinositide-dependent kinase; PIP_2_, phosphatidylinositol 4,5-bisphosphate; PIP_3,_ phosphatidylinositol 3,4,5-trisphosphate; TSCL1/2, T-cell leukemia 1 and 2; GSK, glycogen synthase kinase; BAD, Bcl-2-associated death promoter; FOXO, forkhead box, O subclass; MDM2, mouse double minute 2 homolog; SHC, Src homology 2 domain-containing; GRB2, growth factor receptor-bound protein 2; SOS, son of sevenless; RAS, retrovirus-associated DNA sequences; RAF, rapidly accelerated fibrosarcoma; MEK, mitogen-activated protein kinase; ERK, extracellular signal–regulated kinases.

*PIK3CA* is a part of the phosphatidylinositol 3-kinase (PI3K)/protein kinase B (AKT)/mammalian target of rapamycin (mTOR) pathway, a very commonly disrupted pathway observed across several cancer sites that is involved in the regulation of cell growth, proliferation, differentiation, glucose metabolism, protein synthesis, and apoptosis [96,97,98,99] (Figure 4).

*PIK3CA* encodes p110α, the catalytic subunit of PI3K, and is considered an oncogene; mutations and copy number variations of *PIK3CA* and other related genes in this pathway can contribute to unchecked growth, invasion, and metastasis [100]. *PIK3CA* is the most frequently mutated gene in HPV-positive cancers, with frequencies ranging from 6 to 42% in cervical squamous cell carcinoma, 10–42% in cervical adenocarcinoma, and 22–56% in HPV-positive head and neck cancers [2,18,35,36,37,60,61,62,63,64,65,66,67,68,69]. 

The most common *PIK3CA* mutations occur in “hotspots” E542K and E545K in the helical domain (exon 9) of p110α. Mutations in these sites have been shown to increase phosphatidylinositol 3,4,5-trisphosphate (PIP_3_) levels, activate downstream effectors such as phosphoinositide-dependent kinase (PDK1) and AKT, and promote cellular transformation. Although the mechanisms by which these mutations activate P13K signaling are not fully understood, current data suggests these mutations block the inhibitory effect of the p85α regulatory subunit on p110α activity [101]. In HPV-positive head and neck and cervical squamous cancers, mutations in *PIK3CA* are almost exclusively found in E542K (c.1624G > A) and E545K (1633G > A) corresponding to a C to T single base change at a TCW motif, indicative of APOBEC-induced mutagenesis [35,53,102,103,104,105]. In contrast, these mutations are less common in HPV-negative head and neck cancers, suggesting that APOBEC activity is the major source of *PIK3CA* mutations in HPV-driven carcinogenesis. Evidence from a limited number of studies suggests that these mutations may represent a late event in cervical carcinogenesis [63,67,105]; however, a comprehensive deep-sequencing study of cervical precancers has not been conducted. 

PTEN is a cell cycle regulator that inhibits rapid cell growth and functions as a tumor suppressor [106]. Signaling of the PI3K pathway is regulated by PTEN through dephosphorylation of PIP_3_ (Figure 4) [107]. *PTEN* mutations are less frequent than *PIK3CA* mutations but are found in 6–13% of cervical carcinomas and 6–10% of HPV-positive head and neck cancers [2,18,35,36]. High rates of concurrent *PIK3CA* mutations with *PTEN* loss have been documented in HPV-positive tumors, ranging from 24 to 56% in head and neck cancers to over 80% in anal cancers [99,108]. In the context of *PTEN* loss or deficiency, helical mutations in *PIK3CA* have been shown to induce tumorigenesis through AKT-dependent signaling; whereas in tumors with intact *PTEN*, helical mutations in *PIK3CA* have been shown to promote cell growth and transformation through AKT-independent pathways involving *PDK1* and its substrate serine/threonine protein kinase family member 3 (*SGK3*) [109]. 

Overall, more than 50% of cancers of the cervix and anus have at least one mutation in the PI3K/AKT pathway [110]. Similarly, mutations in this pathway have been reported in 61% of HPV-positive head and neck cancers (and a similar number of HPV-negative head and neck cancers) [2]. The average across all solid tumors was 38%, suggesting that compared with the known driver mutations in other cancers, PI3K pathway alterations are uniquely high in HPV-driven cancers [110]. It is interesting to note that *PIK3CA* is also commonly mutated in endometrial and some ovarian cancers [111,112], which could make it a hallmark of gynecological cancers as well as of HPV-driven cancers.

### 3.3. Human Leukocyte Antigen

**Figure 5:**
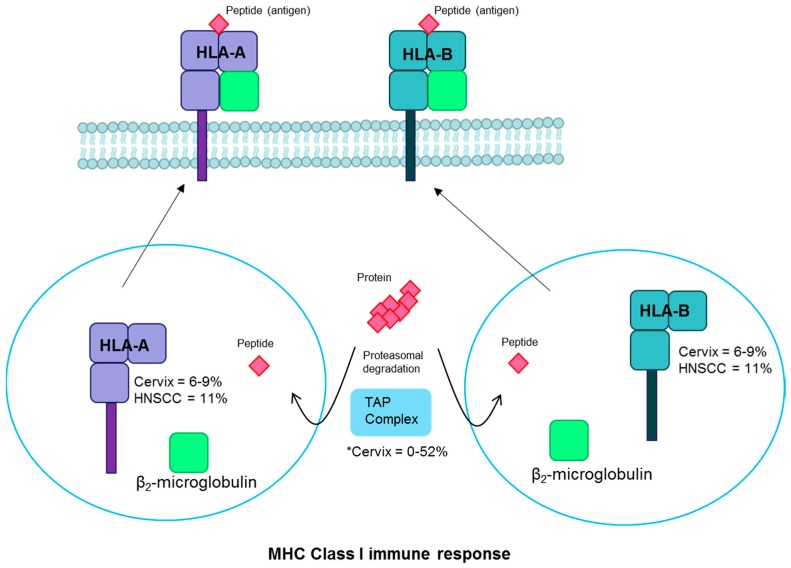
HLA pathway. Proteins undergo proteasomal degradation and the resulting peptides are transported to the endoplasmic reticulum by the TAP complex. There they are bound with MHC Class I into HLA-A or HLA-B and bound to β_2_-microglobulin. The complex is transported to the plasma membrane, where the peptide antigen is displayed for cytotoxic T-cell recognition. Fraction of cervical and head and neck cancers with each gene mutated are noted [2,18,115,116]. * There are conflicting reports of TAP mutation prevalence [115,116]. Abbreviations: MHC, major histocompatibility complex; HLA, human leukocyte antigen; TAP, transporter associated with antigen processing; HNSCC, head and neck squamous cell carcinoma.

*Human leukocyte antigen* (*HLA*) alleles are important components of host cell-mediated immune responses to viral infections and are essential to the major histocompatibility complex (MHC) immune response pathway. *HLA-A* and *HLA-B* are MHC class I molecules that present viral antigens on the cell surface to alert the immune system to infection [113] (Figure 5). Germline HLA variants have been associated with cervical cancer and with HPV-positive oropharyngeal cancer susceptibility [19,20,21]. Somatic mutations are found in *HLA-A* in 8% and *HLA-B* in 6–9% of cervical squamous cell carcinomas [18,35]. In a small study evaluating cervical cancer cell suspensions, 90% of tumors showed some *HLA* gene alterations including gene mutations, loss of heterozygosity, and other genetic changes [78]. *HLA* alterations are found frequently in cervical precancers as well, suggesting that it is an early event in cervical carcinogenesis [114]. Rates of *HLA-A/B* mutations are somewhat more common in HPV-positive (11%) than HPV-negative (7%) head and neck cancers [2,37]. Loss of *HLA-A* or *HLA-B* could lead to loss of presentation of tumor antigens and immune cell recognition. One small study reported frequent mutations in the HLA pathway-associated transporter associated with antigen processing (*TAP*) gene (52%) in cervical carcinomas [115]. However, another candidate gene study failed to replicate this finding [116] and the large cervical cancer studies did not identify recurrent mutations in this gene [18,35]. Given the observed associations of both germline and somatic changes with the antigen presentation pathway, it is clear that it plays an important role in the host response to viral invasion that can alter the probability of persistence and potentially subsequent steps in the carcinogenesis process.

### 3.4. Transforming Growth Factor Beta Pathway

**Figure 6:**
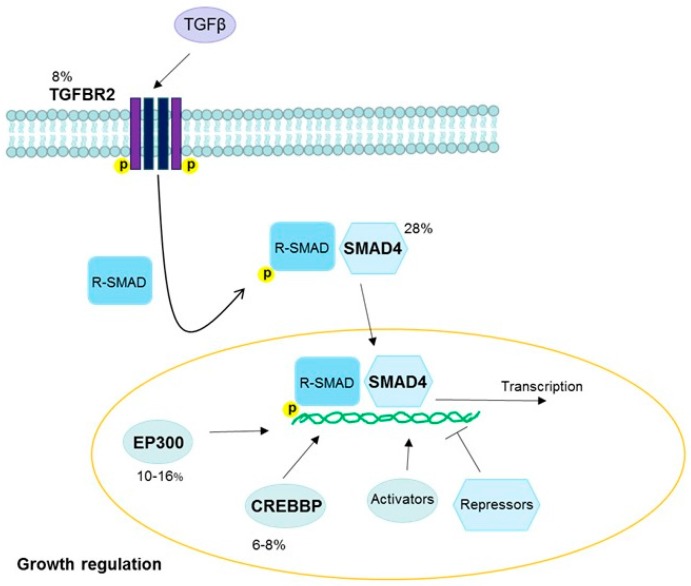
TGFβ pathway. TGFβ binds to TGFBR2 and other receptors to form a complex which becomes phosphorylated. This triggers the phosphorylation of R-SMADs. The phosphorylated R-SMADs form a complex with SMAD4 and are transported into the nucleus, where they promote transcription by binding to promotor regions of the DNA. EP300 and CREBBP are two activators commonly mutated in HPV-driven cancers, and many other activators and repressors also act to regulate this pathway. Fractions of cervical cancers with each gene mutated are noted [18]. Abbreviations: TGFβ, transforming growth factor beta; TGFBR2, TGFβ receptor 2; R-SMAD, receptor-regulated SMAD; EP300, E1A binding protein p300; CREBBP, CREB binding protein.

The transforming growth factor beta (TGFβ) pathway inhibits DNA synthesis and plays a tumor suppressor role, although it can also promote cancer progression once carcinogenesis has been initiated [117,118,119]. Inhibition of this pathway by the HPV oncoprotein E7 contributes to early tumor development in HPV-positive cervical and head and neck cancers [120,121,122,123] (Figure 6). Commonly mutated genes in the TGFβ pathway include *TGFBR2* (a receptor), CREB binding protein (*CREBBP*) and E1A binding protein p300 (*EP300*) (activators), and *SMAD4* (a transcription factor and tumor suppressor), and mutations in at least one of these genes have been reported in 30% of cervical squamous cell carcinomas [18]. In contrast, among TGFβ genes, only *EP300* was in the top 30 mutated genes in head and neck squamous cell carcinomas [36]. This implies that somatic alterations in *TGFBR2*, *CREBBP*, and *SMAD4* may be cervical squamous cell carcinoma-specific, although E7-driven expression effects in the TGFβ pathway may still play a role in carcinogenesis in other HPV-positive cancers. *SMAD4* downregulation is also associated with HPV-negative head and neck cancers [124], and SMAD signaling pathway alterations have been found in both HPV-positive and HPV-negative tumors [37]. 

### 3.5. Notch Pathway

The Notch signaling pathway is responsible for cellular differentiation. Mutations in the *NOTCH1* receptor are found in both HPV-negative (12–26%) and in HPV-positive (6–17%) head and neck cancers, albeit somewhat more frequently in HPV-negative tumors, and are not commonly reported in cervix or other HPV-driven cancer sites [2,36,37,76,77]. This mutation may, therefore, be specific to head and neck carcinogenesis rather than to HPV infection, and *NOTCH1* has indeed been reported as a driver gene in oral tumorigenesis independent of HPV status [125]. F-box and WD repeat domain containing 7 (*FBXW7*) is involved in angiogenesis through regulation of the Notch pathway [126] and is mutated at higher rates in cervix (11–15%) and HPV-positive head and neck (12%) squamous cell carcinomas than in combined head and neck squamous cell carcinomas (HPV status not specified) (5%) [18,35,36].

### 3.6. RAS/EGFR/ERK Pathway

The RAS/EGFR/ERK (retrovirus-associated DNA sequences/ epidermal growth factor receptor/ extracellular signal–regulated kinases) pathway is involved in cellular proliferation and survival (Figure 3). It consists of a signaling cascade that regulates transcription of genes affecting many functions including differentiation, growth, and senescence, which can contribute to carcinogenesis [127]. KRAS proto-oncogene, GTPase (*KRAS*) is an oncogene in which mutations are found in 8–23% in cervical adenocarcinomas but rarely in cervical squamous cell carcinomas [18,35,62,75]. The mutation rate of *KRAS* in head and neck cancers is 6% [37]. In contrast, *EGFR* is a tumor suppressor in the same pathway in which mutations are found in 3–33% of cervical squamous cell carcinomas but rarely in cervical adenocarcinomas [18,62,70,71]. Other genes in this pathway are mutated in fewer than 10% of HPV-positive tumors except for *FGFR2* and *FGFR3*, which have combined mutation rates of 10–17% in HPV-positive head and neck cancers [2,18,35,36,37]. This is notable because, as kinases, the *FGFR* genes may potentially be therapeutic targets [37]. 

### 3.7. Other Genes

The tumor necrosis factor (TNF) receptor associated factor 3 (*TRAF3*) is involved in viral immune responses [128] and was recently reported to have truncating mutations (8%) or deletions (14%) in HPV-positive head and neck cancers [2]. It is not commonly mutated in cervical cancers [18], and it remains to be investigated whether this gene is mutated in HPV-positive cancers at other sites. Other genes differentially mutated in HPV-positive versus HPV-negative head and neck squamous cell carcinomas include *E2F1*, a cell cycle related gene more commonly mutated in HPV-positive cancers (19% versus 2%), and FAT atypical cadherin 1 (*FAT1*) and ajuba LIM protein (*AJUBA*), two genes involved in differentiation that are more commonly mutated in HPV-negative cancers (32% versus 3% and 7% versus 0%, respectively) [2]. 

## 4. Discussion

While HPV infection is a necessary cause of many cancers, the interplay between the virus and the host cell is what ultimately causes cancers to develop. There are many similarities across sites in the mechanisms and mutations found in HPV-driven cancers, suggesting that mechanisms are likely to be similar in rarer cancers such as penile and vaginal carcinomas in which it is difficult to complete large genomic studies. For example, one recent candidate gene study found no statistically significant differences in gene mutations in any of 48 candidate genes including *PIK3CA*, *EGFR*, *NOTCH1*, and *KRAS* or copy number alterations in any of six candidate genes across HPV-positive squamous cell carcinomas at four anatomical sites [99]. While HPV-positive cancers share many characteristic mutagenesis mechanisms and somatic mutations, there are also site-specific aspects. The other major gynecological cancers, endometrial and ovarian cancer, share with cervical cancer high rates of *PIK3CA* mutations and APOBEC and signature 1B mutational signatures. HPV-positive and HPV-negative tumors arising in the head and neck also share properties such as recurrent focal amplifications of the 3q26-28 chromosomal region. Recent data have shown that HPV genetic variation is very common and that HPV variant sublineages influence the risk of different histologic types of cervical precancer and cancer. It will be important to study the interplay between viral genetics and host genomic changes to better understand HPV-driven carcinogenesis [129,130]. 

Characterizing somatic mutations in HPV-related carcinogenesis could be highly relevant for early detection, prognosis, and treatment. To date, very few studies have attempted to characterize the somatic landscape of precancerous lesions, none comprehensively [63,74,131]. Several important steps are required to develop early detection assays based on somatic mutations. First, the sequence of somatic mutation events in the transition from precancers to cancers needs to be established. Next, a promising panel of mutations needs to be selected and evaluated in cervical cytology samples. Similar efforts have been evaluated for other gynecological cancers [132]. 

In addition to early detection, somatic characterization can be important for prognosis and targeted treatment strategies. For example, *PIK3CA*-mutated cervical cancers have worse prognosis than cancer with wild-type *PIK3CA* [61]. Site-specific mutations in *PIK3CA* have been shown to have varying responses to treatment, with evidence suggesting a greater response to PI3K/AKT/mTOR pathway inhibitors for tumors with mutations in the H1047R kinase domain (which are not commonly found in cervical cancers) compared with mutations at other sites [133]. Another prospective therapeutic target is *BCAR4*, in which amplifications and gene fusions have been found in cervical cancer and which is targeted by lapatinib [18,134]. *CD274* and *PDCD1LG2* are immunotherapy targets with amplifications reported in cervical cancer [18]. Erb-b2 receptor tyrosine kinase 2 (*ERBB2; HER2*) and erb-b2 receptor tyrosine kinase 3 (*ERBB3*; *HER3*) are mutated in a subset of cervical adenocarcinomas and these tumors may be susceptible to targeted therapies, and *PTEN* and AT-rich interaction domain 1A (*ARID1A*) alterations are also potential targets [18]. The PI3K/AKT and TGFβ signaling pathways, at least one of which is altered in over 70% of cervical cancers, are very promising in that targeted therapies may be broadly applicable due to their high prevalence [18]. The development of somatic marker panels for HPV-driven cancers will enable oncologists to more precisely tailor treatments.

## Figures and Tables

**Table 1:** Common copy number alterations in HPV-driven cancers.

Arm or Location ^1^	Gene	Pathway	Alteration	Cancer Site ^2^	Fraction Altered in HPV+ Cancers (%)	References
1p	--	--	Gain	Cervix squamous	33	[35]
1q	--	--	Gain	Cervix squamous	29–36	[33,35]
	--	--	Gain	Cervix adeno	22–35	[33,35]
2q	--	--	Loss	Cervix squamous	22	[33]
3p	--	--	Loss	Cervix squamous	36–51	[33,34,35]
	--	--	Loss	Vulva	45	[33]
3p24.1	*TGFBR2*	TGFβ	Gain	Cervix	36	[18]
3p14.1	*FOXP1*	Transcription	Loss	Cervix squamous	42	[34]
3q	--	--	Gain	Cervix	66	[18,33,35]
	--	--	Gain	Cervix squamous	44–62	[33,34,35]
	--	--	Gain	Cervix adeno	29–39	[33,35]
	--	--	Gain	Vulva	58	[33]
3q25.32	*MLF1*	Phenotypic determination	Gain	Cervix squamous	60	[34]
3q26.32	*PIK3CA*	PI3K/AKT	Gain	Head and neck	30–56	[2,36]
3q26.33	*SOX2*	Transcription-Sox2	Gain	Head and neck	11–28	[2,36]
3q27.1	*KLHL6*	Immune signaling	Gain	Head and neck	1–25	[2,36]
3q27.3	*BCL6*	RTK–JAK–STAT	Gain	Head and neck	1–25	[2,36]
3q28	*TP63*	p53	Gain	Cervix	77	[18]
3q28	*LPP*	Cell-cell adhesion	Gain	Cervix squamous	60	[34]
4p	--	--	Loss	Cervix squamous	24–47	[33,35]
	--	--	Loss	Cervix adeno	10–46	[33,35]
	--	--	Loss	Vulva	27	[33]
4q	--	--	Loss	Cervix squamous	21–34	[33,35]
	--	--	Loss	Cervix adeno	17–42	[33,35]
4q31.3	*FBXW7*	Notch	Loss	Head and neck	3–12	[2,36]
5p	--	--	Gain	Cervix squamous	27–28	[33,35]
	--	--	Gain	Vulva	15	[33]
5p13.1	*RICTOR*	PI3K/AKT	Gain	Head and neck	4–6	[2,36]
6p	--	--	Loss	Cervix squamous	24	[35]
6q	--	--	Loss	Cervix squamous	20–29	[33,35]
7p	*EGFR*	RAS/EGFR/ERK	Gain	Cervix	17	[18]
8p	--	--	Loss	Cervix squamous	27	[35]
8q	--	--	Gain	Cervix squamous	25	[34,35]
8q24.21	*MYC*	TGFβ	Gain	Head and neck	3–6	[2,36]
9p	*CD274*	Immune response	Gain	Cervix	8	[18]
10q23.31	*PTEN*	PI3K/AKT	Loss	Cervix	31	[2,18,36]
			Loss	Head and neck	3–15	[2,36]
11p	--	--	Loss	Cervix squamous	32	[35]
	--	--	Loss	Cervix adeno	35	[35]
11q	--	--	Loss	Cervix squamous	32–33	[33,35]
	--	--	Loss	Cervix adeno	9–35	[33,35]
	--	--	Loss	Vulva	30	[33]
11q13.3	*FGF19*	RAS/EGFR/ERK	Gain	Head and neck	4–6	[2,36]
11q13.3	*FGF3*	RAS/EGFR/ERK	Gain	Head and neck	4–6	[2,36]
11q13.3	*FGF4*	RAS/EGFR/ERK	Gain	Head and neck	4–6	[2,36]
11q22.1	*YAP1*	Hippo	Gain	Cervix	16	[18]
13q	--	--	Loss	Cervix squamous	24–41	[33,35]
	--	--	Loss	Cervix adeno	21	[33]
	--	--	Loss	Vulva	12	[33]
13q14.2	*RB1*	Rb	Loss	Head and neck	6–24	[2,36]
14q	--	--	Gain	Cervix squamous	26	[35]
14q32.32	*TRAF3*	NF-κB	Loss	Head and neck	14	[2]
14q32.33	*AKT1*	PI3K/AKT	Gain	Head and neck	5	[2]
16p	--	--	Loss	Cervix adeno	33	[35]
16p13.13	*BCAR4*	Hedgehog	Gain	Cervix	7	[18]
16q	--	--	Loss	Cervix adeno	45	[35]
17p	--	--	Loss	Cervix squamous	34	[35]
18q	--	--	Loss	Cervix adeno	54	[35]
18q21.2	*SMAD4*	TGFβ	Gain	Cervix	28	[18]
19p	--	--	Loss	Cervix adeno	30	[35]
19q	--	--	Gain	Cervix squamous	23	[35]
	--	--	Gain	Cervix adeno	32	[35]
20p	--	--	Gain	Cervix squamous	33	[35]
	--	--	Gain	Cervix adeno	26	[35]
20q	--	--	Gain	Cervix squamous	31	[35]
Xp11.3	*KDM6A*	Chromatin organization	Loss	Head and neck	3–7	[2,36]

^1^ Ordered by chromosome arm or location. ^2^ Cervix is listed when references did not differentiate between squamous cell carcinoma and adenocarcinoma. Sites other than cervix always refer to squamous cell carcinoma. Abbreviations: Adeno, adenocarcinoma; TGFBR2, transforming growth factor beta receptor 2; TGFβ, transforming growth factor beta; FOXP1, forkhead box P1; MLF1, myeloid leukemia factor 1; PIK3CA, phosphatidylinositol-4,5-bisphosphate 3-kinase catalytic subunit alpha; PI3K/AKT, phosphatidylinositol 3-kinase/protein kinase B; SOX2, SRY-box 2; KLHL6, kelch like family member 6; BCL6, B-cell CLL/lymphoma 6; RTK-JAK–STAT, RTK, receptor tyrosine kinase-Janus kinase-signal transducer and activator of transcription; TP63, tumor protein p63; LPP, LIM domain containing preferred translocation partner in lipoma; FBXW7, F-box and WD repeat domain containing 7; RICTOR, RPTOR independent companion of MTOR complex 2; EGFR, epidermal growth factor receptor; RAS, retrovirus-associated DNA sequences; ERK, extracellular signal-regulated kinases; MYC, MYC proto-oncogene, bHLH transcription factor; PTEN, phosphatase and tensin homolog; FGF19, fibroblast growth factor 19; FGF3, fibroblast growth factor 3; FGF4, fibroblast growth factor 4; YAP1, Yes associated protein 1; RB1, RB transcriptional corepressor 1; Rb, retinoblastoma; TRAF3, TNF receptor associated factor 3; NF-κB, nuclear factor kappa-light-chain-enhancer of activated B cells; AKT1, AKT serine/threonine kinase 1; BCAR4, breast cancer anti-estrogen resistance 4; SMAD4, SMAD family member 4; KDM6A, lysine demethylase 6A.

**Table 2:** Common Somatic Mutations in HPV-Driven Cancers.

Gene ^1^	Pathway	Mutation	Cancer Site ^2^	Fraction Mutated in HPV+ Cancers (%)	References
*PIK3CA* ^3^	PI3K/AKT	Activating	Cervix squamous	6–42	[18,35,60,61,62,63,64,65,66,67,68,69]
			Cervix adeno	10–42	[18,35,60,61,62,63,64,65,66,67,68,69]
			Head and neck	22–56	[2,36,37]
*EGFR*	RAS/EGFR/ERK	Activating	Cervix squamous	3–33	[18,62,70,71]
			Cervix adeno	6	[18,62,70]
*SMAD4*	TGFβ	Inactivating	Cervix	28	[18]
*ERBB2*	PI3K/AKT	Activating	Cervix squamous	4	[18]
			Cervix adeno	26	[18]
*TP53*	DNA repair	Inactivating	Cervix squamous	5	[35]
			Head and neck	0–25	[2,3,36,37,72]
			Vulvar	8–16	[73,74]
*RB1*	Rb	Inactivating	Head and neck	6–24	[2,36,37]
*FGFR2 & FGFR3*	RAS/EGFR/ERK	Activating	Head and neck	1–24	[2,36,37]
*KRAS*	RAS/EGFR/ERK	Activating	Cervix squamous	4	[18,35,62]
			Cervix adeno	8–23	[18,35,62,75]
			Head and neck	6	[37]
*MLL2*	Chromatin organization	Activating	Head and neck	10–20	[2,36]
*ASXL1*		Inactivating	Head and neck	5–19	[36]
*NOTCH1*	Notch	Activating, inactivating	Head and neck	6–18	[2,36,37,76,77]
*EP300*	TGFβ	Inactivating	Cervix squamous	13–16	[18]
			Cervix adeno	10	[18]
			Head and neck	10–14	[36]
*ERBB3*	PI3K/AKT	Activating	Cervix squamous	4	[18]
			Cervix adeno	16	[18]
*ATM*	DNA repair	Inactivating	Head and neck	1–16	[36]
*FBXW7*	Notch	Inactivating	Cervix	11–15	[18,35]
			Head and neck	3–12	[36]
*PTEN*	PI3K/AKT	Inactivating	Cervix squamous	6–13	[18,35]
			Cervix adeno	13	[18,35]
			Head and neck	3–15	[2,36]
*BRCA1*	DNA repair	Inactivating	Head and neck	2–14	[36]
*NF1*	RAS/EGFR/ERK	Inactivating	Head and neck	0–14	[2,36]
*ELF3*	PI3K/AKT	Inactivating	Cervix adeno	13	[35]
*FLG*			Head and neck	12	[2]
*BRCA2*	DNA repair	Inactivating	Head and neck	3–12	[36]
*LRP1B*	RTK		Head and neck	2–12	[36]
*HRAS*	RAS/EGFR/ERK	Activating	Head and neck	1–12	[36]
*HLA-A/B*	MHC	Inactivating	Cervix	6–9	[18,35,78]
			Head and neck	11	[2,37]
*MLL3*	Chromatin organization	Activating	Head and neck	10	[2]
*TGFBR2*	TGFβ	Inactivating	Cervix squamous	8	[18]
*CREBBP*	TGFβ	Inactivating	Cervix squamous	8	[18]
			Cervix adeno	6	[18]
*TRAF3*	NF-κB	Truncating mutations	Head and neck	8	[2]
*MAPK1*	MEK/ERK	Activating	Cervix squamous	8	[18,35]
*CBFB*	RUNX1/RUNX2	Inactivating	Cervix adeno	8	[35]
*DDX3X*		Inactivating	Head and neck	8	[2]
*ARID1A*	Chromatin organization	Inactivating	Cervix	7	[18]
*NFE2L2*		Inactivating	Cervix squamous	4–7	[18,35]
*TPRX1*			Head and neck	6	[2]
*CYLD*	NF-κB	Inactivating	Head and neck	6	[2]
*RIPK4*	NF-κB, Notch		Head and neck	6	[2]
*UBR5*	Proteolysis		Head and neck	6	[2]
*CASP8*	Fas apoptosis	Inactivating	Cervix	4	[18]
*STK11*	PI3K/AKT	Inactivating	Cervix squamous	4	[35]
*SHKBP1*	RAS/EGFR/ERK		Cervix	2	[18]

^1^ Ordered by higher reported fraction mutated. ^2^ Cervix is listed when references did not differentiate between squamous cell carcinoma and adenocarcinoma. Sites other than cervix always refer to squamous cell carcinoma. ^3^ Significantly mutated genes in TCGA cervical cancer data are bolded [18]. Abbreviations: ERBB2, erb-b2 receptor tyrosine kinase 2; KRAS, KRAS proto-oncogene, GTPase; MLL2, lysine methyltransferase 2D; ASXL1, additional sex combs like 1, transcriptional regulator; EP300, E1A binding protein p300; ERBB3, erb-b2 receptor tyrosine kinase 2; ATM, ATM serine/threonine kinase; BRCA1, BRCA1, DNA repair associated; NF1, neurofibromin 1; ELF3, E74 like ETS transcription factor 3; FLG, filaggrin; BRCA2, BRCA2, DNA repair associated; LRP1B, LDL receptor related protein 1B; HRAS, HRas proto-oncogene, GTPase; HLA-A/B, human leukocyte antigen A/B; MHC, major histocompatibility complex; MLL3, lysine methyltransferase 2C; CREBBP, CREB binding protein; MAPK1, mitogen-activated protein kinase 1; CBFB, core-binding factor beta subunit; RUNX1/RUNX2, runt related transcription factor 1/2, DDX3X, DEAD-box helicase 3, X-linked; ARID1A, AT-rich interaction domain 1A; NFE2L2, nuclear factor, erythroid 2 like 2; TPRX1, tetrapeptide repeat homeobox 1; CYLD, CYLD lysine 63 deubiquitinase; RIPK4, receptor interacting serine/threonine kinase 4; UBR5, ubiquitin protein ligase E3 component n-recognin 5; CASP8, caspase 8; STK11, serine/threonine kinase 11; SHKBP1, SH3KBP1 binding protein 1.

## References

[1] Walboomers J.M., Jacobs M.V., Manos M.M., Bosch F.X., Kummer J.A., Shah K.V., Snijders P.J., Peto J., Meijer C.J., Munoz N. (1999). Human papillomavirus is a necessary cause of invasive cervical cancer worldwide. J. Pathol..

[2] Cancer Genome Atlas Network (2015). Comprehensive genomic characterization of head and neck squamous cell carcinomas. Nature.

[3] Stransky N., Egloff A.M., Tward A.D., Kostic A.D., Cibulskis K., Sivachenko A., Kryukov G.V., Lawrence M.S., Sougnez C., McKenna A. (2011). The mutational landscape of head and neck squamous cell carcinoma. Science.

[4] Ndiaye C., Mena M., Alemany L., Arbyn M., Castellsague X., Laporte L., Bosch F.X., de Sanjose S., Trottier H. (2014). HPV DNA, E6/E7 mRNA, and p16^INK4A^ detection in head and neck cancers: A systematic review and meta-analysis. Lancet Oncol..

[5] Hartwig S., Baldauf J.-J., Dominiak-Felden G., Simondon F., Alemany L., de Sanjosé S., Castellsagué X. (2015). Estimation of the epidemiological burden of HPV-related anogenital cancers, precancerous lesions, and genital warts in women and men in europe: Potential additional benefit of a nine-valent second generation HPV vaccine compared to first generation HPV vaccines. Papillomavirus Res..

[6] Backes D.M., Kurman R.J., Pimenta J.M., Smith J.S. (2009). Systematic review of human papillomavirus prevalence in invasive penile cancer. Cancer Causes Control.

[7] Miralles-Guri C., Bruni L., Cubilla A.L., Castellsague X., Bosch F.X., de Sanjose S. (2009). Human papillomavirus prevalence and type distribution in penile carcinoma. J. Clin. Pathol..

[8] Forman D., de Martel C., Lacey C.J., Soerjomataram I., Lortet-Tieulent J., Bruni L., Vignat J., Ferlay J., Bray F., Plummer M. (2012). Global burden of human papillomavirus and related diseases. Vaccine.

[9] De Martel C., Ferlay J., Franceschi S., Vignat J., Bray F., Forman D., Plummer M. (2012). Global burden of cancers attributable to infections in 2008: A review and synthetic analysis. Lancet Oncol..

[10] Ho G.Y., Bierman R., Beardsley L., Chang C.J., Burk R.D. (1998). Natural history of cervicovaginal papillomavirus infection in young women. N. Engl. J. Med..

[11] Schiffman M., Kjaer S.K. (2003). Chapter 2: Natural history of anogenital human papillomavirus infection and neoplasia. J. Natl. Cancer Inst. Monogr..

[12] Rositch A.F., Koshiol J., Hudgens M.G., Razzaghi H., Backes D.M., Pimenta J.M., Franco E.L., Poole C., Smith J.S. (2013). Patterns of persistent genital human papillomavirus infection among women worldwide: A literature review and meta-analysis. Int. J. Cancer.

[13] Moscicki A.B., Schiffman M., Kjaer S., Villa L.L. (2006). Chapter 5: Updating the natural history of HPV and anogenital cancer. Vaccine.

[14] McCredie M.R., Sharples K.J., Paul C., Baranyai J., Medley G., Jones R.W., Skegg D.C. (2008). Natural history of cervical neoplasia and risk of invasive cancer in women with cervical intraepithelial neoplasia 3: A retrospective cohort study. Lancet Oncol..

[15] Schiffman M., Doorbar J., Wentzensen N., de Sanjose S., Fakhry C., Monk B.J., Stanley M.A., Franceschi S. (2016). Carcinogenic human papillomavirus infection. Nat. Rev. Dis. Primers.

[16] Wentzensen N., Arbyn M., Berkhof J., Bower M., Canfell K., Einstein M., Farley C., Monsonego J., Franceschi S. (2017). Eurogin 2016 roadmap: How HPV knowledge is changing screening practice. Int. J. Cancer.

[17] Rusan M., Li Y.Y., Hammerman P.S. (2015). Genomic landscape of human papillomavirus-associated cancers. Clin. Cancer Res..

[18] The Cancer Genome Atlas Research Network (2017). Integrated genomic and molecular characterization of cervical cancer. Nature.

[19] Chen D., Gyllensten U. (2015). Lessons and implications from association studies and post-gwas analyses of cervical cancer. Trends Genet..

[20] Martínez-Nava G.A., Fernández-Niño J.A., Madrid-Marina V., Torres-Poveda K. (2016). Cervical cancer genetic susceptibility: A systematic review and meta-analyses of recent evidence. PLoS ONE.

[21] Lesseur C., Diergaarde B., Olshan A.F., Wunsch-Filho V., Ness A.R., Liu G., Lacko M., Eluf-Neto J., Franceschi S., Lagiou P. (2016). Genome-wide association analyses identify new susceptibility loci for oral cavity and pharyngeal cancer. Nat. Genet..

[22] Li N., Franceschi S., Howell-Jones R., Snijders P.J., Clifford G.M. (2011). Human papillomavirus type distribution in 30,848 invasive cervical cancers worldwide: Variation by geographical region, histological type and year of publication. Int. J. Cancer.

[23] Prigge E.S., von Knebel Doeberitz M., Reuschenbach M. (2017). Clinical relevance and implications of HPV-induced neoplasia in different anatomical locations. Mutat. Res..

[24] Duensing S., Munger K. (2001). Centrosome abnormalities, genomic instability and carcinogenic progression. Biochim. Biophys. Acta.

[25] Duensing S., Lee L.Y., Duensing A., Basile J., Piboonniyom S., Gonzalez S., Crum C.P., Munger K. (2000). The human papillomavirus type 16 E6 and E7 oncoproteins cooperate to induce mitotic defects and genomic instability by uncoupling centrosome duplication from the cell division cycle. Proc. Natl. Acad. Sci. USA.

[26] Werness B.A., Levine A.J., Howley P.M. (1990). Association of human papillomavirus types 16 and 18 E6 proteins with p53. Science.

[27] Scheffner M., Werness B.A., Huibregtse J.M., Levine A.J., Howley P.M. (1990). The E6 oncoprotein encoded by human papillomavirus types 16 and 18 promotes the degradation of p53. Cell.

[28] Dyson N., Howley P.M., Munger K., Harlow E. (1989). The human papilloma virus-16 E7 oncoprotein is able to bind to the retinoblastoma gene product. Science.

[29] Munger K., Werness B.A., Dyson N., Phelps W.C., Harlow E., Howley P.M. (1989). Complex formation of human papillomavirus E7 proteins with the retinoblastoma tumor suppressor gene product. EMBO J..

[30] Boyer S.N., Wazer D.E., Band V. (1996). E7 protein of human papilloma virus-16 induces degradation of retinoblastoma protein through the ubiquitin-proteasome pathway. Cancer Res..

[31] McDaniel A.S., Hovelson D.H., Cani A.K., Liu C.J., Zhai Y., Zhang Y., Weizer A.Z., Mehra R., Feng F.Y., Alva A.S. (2015). Genomic profiling of penile squamous cell carcinoma reveals new opportunities for targeted therapy. Cancer Res..

[32] Warren C.J., Xu T., Guo K., Griffin L.M., Westrich J.A., Lee D., Lambert P.F., Santiago M.L., Pyeon D. (2015). APOBEC3A functions as a restriction factor of human papillomavirus. J. Virol..

[33] Thomas L.K., Bermejo J.L., Vinokurova S., Jensen K., Bierkens M., Steenbergen R., Bergmann M., von Knebel Doeberitz M., Reuschenbach M. (2014). Chromosomal gains and losses in human papillomavirus-associated neoplasia of the lower genital tract—A systematic review and meta-analysis. Eur. J. Cancer.

[34] Bodelon C., Vinokurova S., Sampson J.N., den Boon J.A., Walker J.L., Horswill M.A., Korthauer K., Schiffman M., Sherman M.E., Zuna R.E. (2016). Chromosomal copy number alterations and HPV integration in cervical precancer and invasive cancer. Carcinogenesis.

[35] Ojesina A.I., Lichtenstein L., Freeman S.S., Pedamallu C.S., Imaz-Rosshandler I., Pugh T.J., Cherniack A.D., Ambrogio L., Cibulskis K., Bertelsen B. (2014). Landscape of genomic alterations in cervical carcinomas. Nature.

[36] Chung C.H., Guthrie V.B., Masica D.L., Tokheim C., Kang H., Richmon J., Agrawal N., Fakhry C., Quon H., Subramaniam R.M. (2015). Genomic alterations in head and neck squamous cell carcinoma determined by cancer gene-targeted sequencing. Ann. Oncol..

[37] Seiwert T.Y., Zuo Z., Keck M.K., Khattri A., Pedamallu C.S., Stricker T., Brown C., Pugh T.J., Stojanov P., Cho J. (2015). Integrative and comparative genomic analysis of HPV-positive and HPV-negative head and neck squamous cell carcinomas. Clin. Cancer Res..

[38] Busso-Lopes A.F., Marchi F.A., Kuasne H., Scapulatempo-Neto C., Trindade-Filho J.C., de Jesus C.M., Lopes A., Guimaraes G.C., Rogatto S.R. (2015). Genomic profiling of human penile carcinoma predicts worse prognosis and survival. Cancer Prev. Res..

[39] Heselmeyer K., du Manoir S., Blegen H., Friberg B., Svensson C., Schrock E., Veldman T., Shah K., Auer G., Ried T. (1997). A recurrent pattern of chromosomal aberrations and immunophenotypic appearance defines anal squamous cell carcinomas. Br. J. Cancer.

[40] Wentzensen N., Ridder R., Klaes R., Vinokurova S., Schaefer U., Doeberitz M. (2002). Characterization of viral-cellular fusion transcripts in a large series of HPV16 and 18 positive anogenital lesions. Oncogene.

[41] Vinokurova S., Wentzensen N., Kraus I., Klaes R., Driesch C., Melsheimer P., Kisseljov F., Durst M., Schneider A., von Knebel Doeberitz M. (2008). Type-dependent integration frequency of human papillomavirus genomes in cervical lesions. Cancer Res..

[42] Bodelon C., Untereiner M.E., Machiela M.J., Vinokurova S., Wentzensen N. (2016). Genomic characterization of viral integration sites in HPV-related cancers. Int. J. Cancer.

[43] Akagi K., Li J., Broutian T.R., Padilla-Nash H., Xiao W., Jiang B., Rocco J.W., Teknos T.N., Kumar B., Wangsa D. (2014). Genome-wide analysis of HPV integration in human cancers reveals recurrent, focal genomic instability. Genome Res..

[44] Schmitz M., Driesch C., Jansen L., Runnebaum I.B., Durst M. (2012). Non-random integration of the HPV genome in cervical cancer. PLoS ONE.

[45] Wentzensen N., Vinokurova S., von Knebel Doeberitz M. (2004). Systematic review of genomic integration sites of human papillomavirus genomes in epithelial dysplasia and invasive cancer of the female lower genital tract. Cancer Res..

[46] Hu Z., Zhu D., Wang W., Li W., Jia W., Zeng X., Ding W., Yu L., Wang X., Wang L. (2015). Genome-wide profiling of HPV integration in cervical cancer identifies clustered genomic hot spots and a potential microhomology-mediated integration mechanism. Nat. Genet..

[47] Conticello S.G. (2008). The AID/APOBEC family of nucleic acid mutators. Genome Biol..

[48] Roberts S.A., Sterling J., Thompson C., Harris S., Mav D., Shah R., Klimczak L.J., Kryukov G.V., Malc E., Mieczkowski P.A. (2012). Clustered mutations in yeast and in human cancers can arise from damaged long single-strand DNA regions. Mol. Cell.

[49] Simonelli V., Narciso L., Dogliotti E., Fortini P. (2005). Base excision repair intermediates are mutagenic in mammalian cells. Nucleic Acids Res..

[50] Alexandrov L.B., Nik-Zainal S., Wedge D.C., Aparicio S.A., Behjati S., Biankin A.V., Bignell G.R., Bolli N., Borg A., Borresen-Dale A.L. (2013). Signatures of mutational processes in human cancer. Nature.

[51] Burns M.B., Temiz N.A., Harris R.S. (2013). Evidence for APOBEC3b mutagenesis in multiple human cancers. Nat. Genet..

[52] Roberts S.A., Lawrence M.S., Klimczak L.J., Grimm S.A., Fargo D., Stojanov P., Kiezun A., Kryukov G.V., Carter S.L., Saksena G. (2013). An APOBEC cytidine deaminase mutagenesis pattern is widespread in human cancers. Nat. Genet..

[53] Henderson S., Chakravarthy A., Su X., Boshoff C., Fenton T.R. (2014). APOBEC-mediated cytosine deamination links PIK3CA helical domain mutations to human papillomavirus-driven tumor development. Cell Rep..

[54] Feber A., Worth D.C., Chakravarthy A., de Winter P., Shah K., Arya M., Saqib M., Nigam R., Malone P.R., Tan W.S. (2016). CSN1 somatic mutations in penile squamous cell carcinoma. Cancer Res..

[55] Vieira V.C., Leonard B., White E.A., Starrett G.J., Temiz N.A., Lorenz L.D., Lee D., Soares M.A., Lambert P.F., Howley P.M. (2014). Human papillomavirus E6 triggers upregulation of the antiviral and cancer genomic DNA deaminase APOBEC3B. MBio.

[56] Rebhandl S., Huemer M., Greil R., Geisberger R. (2015). AID/APOBEC deaminases and cancer. Oncoscience.

[57] Nik-Zainal S., Alexandrov L.B., Wedge D.C., Van Loo P., Greenman C.D., Raine K., Jones D., Hinton J., Marshall J., Stebbings L.A. (2012). Mutational processes molding the genomes of 21 breast cancers. Cell.

[58] Taylor B.J., Nik-Zainal S., Wu Y.L., Stebbings L.A., Raine K., Campbell P.J., Rada C., Stratton M.R., Neuberger M.S. (2013). DNA deaminases induce break-associated mutation showers with implication of APOBEC3B and 3A in breast cancer kataegis. Elife.

[59] Leonard B., Hart S.N., Burns M.B., Carpenter M.A., Temiz N.A., Rathore A., Vogel R.I., Nikas J.B., Law E.K., Brown W.L. (2013). APOBEC3B upregulation and genomic mutation patterns in serous ovarian carcinoma. Cancer Res..

[60] McIntyre J.B., Wu J.S., Craighead P.S., Phan T., Kobel M., Lees-Miller S.P., Ghatage P., Magliocco A.M., Doll C.M. (2013). PIK3CA mutational status and overall survival in patients with cervical cancer treated with radical chemoradiotherapy. Gynecol. Oncol..

[61] Lou H., Villagran G., Boland J.F., Im K.M., Polo S., Zhou W., Odey U., Juarez-Torres E., Medina-Martinez I., Roman-Basaure E. (2015). Genome analysis of latin american cervical cancer: Frequent activation of the PIK3CA pathway. Clin. Cancer Res..

[62] Wright A.A., Howitt B.E., Myers A.P., Dahlberg S.E., Palescandolo E., Van Hummelen P., MacConaill L.E., Shoni M., Wagle N., Jones R.T. (2013). Oncogenic mutations in cervical cancer: Genomic differences between adenocarcinomas and squamous cell carcinomas of the cervix. Cancer.

[63] Cui B., Zheng B., Zhang X., Stendahl U., Andersson S., Wallin K.L. (2009). Mutation of PIK3CA: Possible risk factor for cervical carcinogenesis in older women. Int. J. Oncol..

[64] Rashmi R., DeSelm C., Helms C., Bowcock A., Rogers B.E., Rader J.L., Grigsby P.W., Schwarz J.K. (2014). Akt inhibitors promote cell death in cervical cancer through disruption of mtor signaling and glucose uptake. PLoS ONE.

[65] Spaans V.M., Trietsch M.D., Crobach S., Stelloo E., Kremer D., Osse E.M., Haar N.T., van Eijk R., Muller S., van Wezel T. (2014). Designing a high-throughput somatic mutation profiling panel specifically for gynaecological cancers. PLoS ONE.

[66] Spaans V.M., Trietsch M.D., Peters A.A., Osse M., Ter Haar N., Fleuren G.J., Jordanova E.S. (2015). Precise classification of cervical carcinomas combined with somatic mutation profiling contributes to predicting disease outcome. PLoS ONE.

[67] Tornesello M.L., Annunziata C., Buonaguro L., Losito S., Greggi S., Buonaguro F.M. (2014). TP53 and PIK3CA gene mutations in adenocarcinoma, squamous cell carcinoma and high-grade intraepithelial neoplasia of the cervix. J. Transl. Med..

[68] Hou M.M., Liu X., Wheler J., Naing A., Hong D., Coleman R.L., Tsimberidou A., Janku F., Zinner R., Lu K. (2014). Targeted PI3K/AKT/mTOR therapy for metastatic carcinomas of the cervix: A phase I clinical experience. Oncotarget.

[69] Chung T.K.H., Cheung T.H., Yim S.F., Yu M.Y., Chiu R.W.K., Lo K.W.K., Lee I.P.C., Wong R.R.Y., Lau K.K.M., Wang V.W. (2017). Liquid biopsy of PIK3CA mutations in cervical cancer in Hong Kong Chinese women. Gynecol. Oncol..

[70] Qureshi R., Arora H., Biswas S., Perwez A., Naseem A., Wajid S., Gandhi G., Rizvi M.A. (2016). Mutation analysis of EGFR and its correlation with the HPV in indian cervical cancer patients. Tumour Biol..

[71] Iida K., Nakayama K., Rahman M.T., Rahman M., Ishikawa M., Katagiri A., Yeasmin S., Otsuki Y., Kobayashi H., Nakayama S. (2011). EGFR gene amplification is related to adverse clinical outcomes in cervical squamous cell carcinoma, making the EGFR pathway a novel therapeutic target. Br. J. Cancer.

[72] Westra W.H., Taube J.M., Poeta M.L., Begum S., Sidransky D., Koch W.M. (2008). Inverse relationship between human papillomavirus-16 infection and disruptive p53 gene mutations in squamous cell carcinoma of the head and neck. Clin. Cancer Res..

[73] Kashofer K., Regauer S. (2017). Analysis of full coding sequence of the TP53 gene in invasive vulvar cancers: Implications for therapy. Gynecol. Oncol..

[74] Trietsch M.D., Nooij L.S., Gaarenstroom K.N., van Poelgeest M.I. (2015). Genetic and epigenetic changes in vulvar squamous cell carcinoma and its precursor lesions: A review of the current literature. Gynecol. Oncol..

[75] Kang S., Kim H.S., Seo S.S., Park S.Y., Sidransky D., Dong S.M. (2007). Inverse correlation between RASSF1A hypermethylation, kras and braf mutations in cervical adenocarcinoma. Gynecol. Oncol..

[76] Tinhofer I., Stenzinger A., Eder T., Konschak R., Niehr F., Endris V., Distel L., Hautmann M.G., Mandic R., Stromberger C. (2016). Targeted next-generation sequencing identifies molecular subgroups in squamous cell carcinoma of the head and neck with distinct outcome after concurrent chemoradiation. Ann. Oncol..

[77] Agrawal N., Frederick M.J., Pickering C.R., Bettegowda C., Chang K., Li R.J., Fakhry C., Xie T.X., Zhang J., Wang J. (2011). Exome sequencing of head and neck squamous cell carcinoma reveals inactivating mutations in NOTCH1. Science.

[78] Koopman L.A., Corver W.E., van der Slik A.R., Giphart M.J., Fleuren G.J. (2000). Multiple genetic alterations cause frequent and heterogeneous human histocompatibility leukocyte antigen class I loss in cervical cancer. J. Exp. Med..

[79] Moody C.A., Laimins L.A. (2010). Human papillomavirus oncoproteins: Pathways to transformation. Nat. Rev. Cancer.

[80] Banister C.E., Liu C., Pirisi L., Creek K.E., Buckhaults P.J. (2017). Identification and characterization of HPV-independent cervical cancers. Oncotarget.

[81] Salam M., Rockett J., Morris A. (1995). The prevalence of different human papillomavirus types and p53 mutations in laryngeal carcinomas: Is there a reciprocal relationship?. Eur. J. Surg. Oncol..

[82] Rajendra S., Wang B., Merrett N., Sharma P., Humphris J., Lee H.C., Wu J. (2016). Genomic analysis of HPV-positive versus HPV-negative oesophageal adenocarcinoma identifies a differential mutational landscape. J. Med. Genet..

[83] Zhang W., Edwards A., Fang Z., Flemington E.K., Zhang K. (2016). Integrative genomics and transcriptomics analysis reveals potential mechanisms for favorable prognosis of patients with HPV-positive head and neck carcinomas. Sci. Rep..

[84] Serup-Hansen E., Linnemann D., Skovrider-Ruminski W., Hogdall E., Geertsen P.F., Havsteen H. (2014). Human papillomavirus genotyping and p16 expression as prognostic factors for patients with american joint committee on cancer stages i to iii carcinoma of the anal canal. J. Clin. Oncol..

[85] Lont A.P., Kroon B.K., Horenblas S., Gallee M.P., Berkhof J., Meijer C.J., Snijders P.J. (2006). Presence of high-risk human papillomavirus DNA in penile carcinoma predicts favorable outcome in survival. Int. J. Cancer.

[86] Djajadiningrat R.S., Jordanova E.S., Kroon B.K., van Werkhoven E., de Jong J., Pronk D.T., Snijders P.J., Horenblas S., Heideman D.A. (2015). Human papillomavirus prevalence in invasive penile cancer and association with clinical outcome. J. Urol..

[87] Bezerra A.L., Lopes A., Santiago G.H., Ribeiro K.C., Latorre M.R., Villa L.L. (2001). Human papillomavirus as a prognostic factor in carcinoma of the penis: Analysis of 82 patients treated with amputation and bilateral lymphadenectomy. Cancer.

[88] Bezerra S.M., Chaux A., Ball M.W., Faraj S.F., Munari E., Gonzalez-Roibon N., Sharma R., Bivalacqua T.J., Burnett A.L., Netto G.J. (2015). Human papillomavirus infection and immunohistochemical p16(INK4A) expression as predictors of outcome in penile squamous cell carcinomas. Hum. Pathol..

[89] Schaal C., Pillai S., Chellappan S.P. (2014). The RB-E2F transcriptional regulatory pathway in tumor angiogenesis and metastasis. Adv. Cancer Res..

[90] Doorbar J., Egawa N., Griffin H., Kranjec C., Murakami I. (2015). Human papillomavirus molecular biology and disease association. Rev. Med. Virol..

[91] Felsani A., Mileo A.M., Paggi M.G. (2006). Retinoblastoma family proteins as key targets of the small DNA virus oncoproteins. Oncogene.

[92] El-Naggar A.K., Westra W.H. (2012). p16 expression as a surrogate marker for HPV-related oropharyngeal carcinoma: A guide for interpretative relevance and consistency. Head Neck.

[93] Klaes R., Friedrich T., Spitkovsky D., Ridder R., Rudy W., Petry U., Dallenbach-Hellweg G., Schmidt D., von Knebel Doeberitz M. (2001). Overexpression of p16^INK4A^ as a specific marker for dysplastic and neoplastic epithelial cells of the cervix uteri. Int. J. Cancer.

[94] Wentzensen N., Fetterman B., Castle P.E., Schiffman M., Wood S.N., Stiemerling E., Tokugawa D., Bodelon C., Poitras N., Lorey T. (2015). p16/KI-67 dual stain cytology for detection of cervical precancer in HPV-positive women. J. Natl. Cancer Inst..

[95] Cuschieri K., Wentzensen N. (2008). Human papillomavirus mRNA and p16 detection as biomarkers for the improved diagnosis of cervical neoplasia. Cancer Epidemiol. Biomark. Prev..

[96] Kennedy S.G., Wagner A.J., Conzen S.D., Jordan J., Bellacosa A., Tsichlis P.N., Hay N. (1997). The PI 3-kinase/AKT signaling pathway delivers an anti-apoptotic signal. Genes Dev..

[97] Klippel A., Escobedo M.A., Wachowicz M.S., Apell G., Brown T.W., Giedlin M.A., Kavanaugh W.M., Williams L.T. (1998). Activation of phosphatidylinositol 3-kinase is sufficient for cell cycle entry and promotes cellular changes characteristic of oncogenic transformation. Mol. Cell Biol..

[98] Ma Y.Y., Wei S.J., Lin Y.C., Lung J.C., Chang T.C., Whang-Peng J., Liu J.M., Yang D.M., Yang W.K., Shen C.Y. (2000). PIK3CA as an oncogene in cervical cancer. Oncogene.

[99] Koncar R.F., Feldman R., Bahassi E.M., Hashemi Sadraei N. (2017). Comparative molecular profiling of HPV-induced squamous cell carcinomas. Cancer Med..

[100] Samuels Y., Ericson K. (2006). Oncogenic PI3K and its role in cancer. Curr. Opin. Oncol..

[101] Liu P., Cheng H., Roberts T.M., Zhao J.J. (2009). Targeting the phosphoinositide 3-kinase pathway in cancer. Nat. Rev. Drug Discov..

[102] Vogt P.K., Kang S., Elsliger M.A., Gymnopoulos M. (2007). Cancer-specific mutations in phosphatidylinositol 3-kinase. Trends Biochem. Sci..

[103] Lui V.W., Hedberg M.L., Li H., Vangara B.S., Pendleton K., Zeng Y., Lu Y., Zhang Q., Du Y., Gilbert B.R. (2013). Frequent mutation of the PI3K pathway in head and neck cancer defines predictive biomarkers. Cancer Discov..

[104] Lechner M., Frampton G.M., Fenton T., Feber A., Palmer G., Jay A., Pillay N., Forster M., Cronin M.T., Lipson D. (2013). Targeted next-generation sequencing of head and neck squamous cell carcinoma identifies novel genetic alterations in HPV+ and HPV− tumors. Genome Med..

[105] Verlaat W., Snijders P.J., van Moorsel M.I., Bleeker M., Rozendaal L., Sie D., Ylstra B., Meijer C.J., Steenbergen R.D., Heideman D.A. (2015). Somatic mutation in PIK3CA is a late event in cervical carcinogenesis. J. Pathol. Clin. Res..

[106] Husain R.S., Ramakrishnan V. (2015). Global variation of human papillomavirus genotypes and selected genes involved in cervical malignancies. Ann. Glob. Health.

[107] Carracedo A., Pandolfi P.P. (2008). The PTEN-PI3K pathway: Of feedbacks and cross-talks. Oncogene.

[108] Feldman R., Gatalica Z., Knezetic J., Reddy S., Nathan C.A., Javadi N., Teknos T. (2016). Molecular profiling of head and neck squamous cell carcinoma. Head Neck.

[109] Vasudevan K.M., Barbie D.A., Davies M.A., Rabinovsky R., McNear C.J., Kim J.J., Hennessy B.T., Tseng H., Pochanard P., Kim S.Y. (2009). AKT-independent signaling downstream of oncogenic PIK3CA mutations in human cancer. Cancer Cell.

[110] Millis S.Z., Ikeda S., Reddy S., Gatalica Z., Kurzrock R. (2016). Landscape of phosphatidylinositol-3-kinase pathway alterations across 19,784 diverse solid tumors. JAMA Oncol..

[111] Shayesteh L., Lu Y., Kuo W.L., Baldocchi R., Godfrey T., Collins C., Pinkel D., Powell B., Mills G.B., Gray J.W. (1999). PIK3CA is implicated as an oncogene in ovarian cancer. Nat. Genet..

[112] Markowska A., Pawalowska M., Lubin J., Markowska J. (2014). Signalling pathways in endometrial cancer. Contemp. Oncol..

[113] Hewitt E.W. (2003). The MHC class I antigen presentation pathway: Strategies for viral immune evasion. Immunology.

[114] Vermeulen C.F., Jordanova E.S., Zomerdijk-Nooijen Y.A., ter Haar N.T., Peters A.A., Fleuren G.J. (2005). Frequent HLA class I loss is an early event in cervical carcinogenesis. Hum. Immunol..

[115] Fowler N.L., Frazer I.H. (2004). Mutations in TAP genes are common in cervical carcinomas. Gynecol. Oncol..

[116] Vermeulen C.F., Jordanova E.S., ter Haar N.T., Kolkman-Uljee S.M., de Miranda N.F., Ferrone S., Peters A.A., Fleuren G.J. (2007). Expression and genetic analysis of transporter associated with antigen processing in cervical carcinoma. Gynecol. Oncol..

[117] Huang J.J., Blobe G.C. (2016). Dichotomous roles of TGF-beta in human cancer. Biochem. Soc. Trans..

[118] Deng W., Tsao S.W., Kwok Y.K., Wong E., Huang X.R., Liu S., Tsang C.M., Ngan H.Y., Cheung A.N., Lan H.Y. (2008). Transforming growth factor beta1 promotes chromosomal instability in human papillomavirus 16 E6E7-infected cervical epithelial cells. Cancer Res..

[119] Zhu H., Luo H., Shen Z., Hu X., Sun L., Zhu X. (2016). Transforming growth factor-beta1 in carcinogenesis, progression, and therapy in cervical cancer. Tumour Biol..

[120] Chang H.S., Lin C.H., Yang C.H., Liang Y.J., Yu W.C. (2010). The human papillomavirus-16 (HPV-16) oncoprotein E7 conjugates with and mediates the role of the transforming growth factor-beta inducible early gene 1 (TIEG1) in apoptosis. Int. J. Biochem. Cell Biol..

[121] Habig M., Smola H., Dole V.S., Derynck R., Pfister H., Smola-Hess S. (2006). E7 proteins from high- and low-risk human papillomaviruses bind to TGF-beta-regulated smad proteins and inhibit their transcriptional activity. Arch. Virol..

[122] Murvai M., Borbely A.A., Konya J., Gergely L., Veress G. (2004). Effect of human papillomavirus type 16 E6 and E7 oncogenes on the activity of the transforming growth factor-beta2 (TGF-beta2) promoter. Arch. Virol..

[123] Lee D.K., Kim B.C., Kim I.Y., Cho E.A., Satterwhite D.J., Kim S.J. (2002). The human papilloma virus E7 oncoprotein inhibits transforming growth factor-beta signaling by blocking binding of the smad complex to its target sequence. J. Biol. Chem..

[124] Cheng H., Fertig E.J., Ozawa H., Hatakeyama H., Howard J.D., Perez J., Considine M., Thakar M., Ranaweera R., Krigsfeld G. (2015). Decreased SMAD4 expression is associated with induction of epithelial-to-mesenchymal transition and cetuximab resistance in head and neck squamous cell carcinoma. Cancer Biol. Ther..

[125] Izumchenko E., Sun K., Jones S., Brait M., Agrawal N., Koch W., McCord C.L., Riley D.R., Angiuoli S.V., Velculescu V.E. (2015). NOTCH1 mutations are drivers of oral tumorigenesis. Cancer Prev. Res..

[126] Izumi N., Helker C., Ehling M., Behrens A., Herzog W., Adams R.H. (2012). FBXW7 controls angiogenesis by regulating endothelial notch activity. PLoS ONE.

[127] Santarpia L., Lippman S.M., El-Naggar A.K. (2012). Targeting the MAPK-RAS-RAF signaling pathway in cancer therapy. Expert Opin. Ther. Targets.

[128] Oganesyan G., Saha S.K., Guo B., He J.Q., Shahangian A., Zarnegar B., Perry A., Cheng G. (2006). Critical role of TRAF3 in the toll-like receptor-dependent and -independent antiviral response. Nature.

[129] Mirabello L., Yeager M., Cullen M., Boland J.F., Chen Z., Wentzensen N., Zhang X., Yu K., Yang Q., Mitchell J. (2016). HPV16 sublineage associations with histology-specific cancer risk using HPV whole-genome sequences in 3200 women. J. Natl. Cancer Inst..

[130] Mirabello L., Yeager M., Yu K., Clifford G., Xiao Y., Zhu B., Cullen M., Boland J.F., Wentzensen N., Nelson C.W. (2017). HPV16 E7 genetic conservation is critical to carcinogenesis. Cell.

[131] Pinto A.P., Miron A., Yassin Y., Monte N., Woo T.Y., Mehra K.K., Medeiros F., Crum C.P. (2010). Differentiated vulvar intraepithelial neoplasia contains TP53 mutations and is genetically linked to vulvar squamous cell carcinoma. Mod. Pathol..

[132] Kinde I., Bettegowda C., Wang Y., Wu J., Agrawal N., Shih Ie M., Kurman R., Dao F., Levine D.A., Giuntoli R. (2013). Evaluation of DNA from the Papanicolaou test to detect ovarian and endometrial cancers. Sci. Transl. Med..

[133] Janku F., Wheler J.J., Naing A., Falchook G.S., Hong D.S., Stepanek V.M., Fu S., Piha-Paul S.A., Lee J.J., Luthra R. (2013). PIK3CA mutation H1047R is associated with response to PI3K/AKT/mTOR signaling pathway inhibitors in early-phase clinical trials. Cancer Res..

[134] Godinho M.F., Wulfkuhle J.D., Look M.P., Sieuwerts A.M., Sleijfer S., Foekens J.A., Petricoin E.F., Dorssers L.C., van Agthoven T. (2012). BCAR4 induces antioestrogen resistance but sensitises breast cancer to lapatinib. Br. J. Cancer.

